# Books Reviewed

**Published:** 1869-03

**Authors:** 


### Books Reviewed.

A Treatise upon the Diseases of Infancy and Childhood. By J. Lewis Smith, M. D. Philadelphia: Henry C. Lea, 1869.

The intention of the author has been to present a description of the diseases of infancy and childhood in a sufficiently succinct manner to meet the requirements of practitioners and medical students. He has incorporated into the treatise all recently ascertained facts in this branch of medical practice, and recommended such modes of treatment as are suggested by our present knowledge of the nature of the diseases of early life. He bases his opinions largely upon his own observation, which has been sufficiently ample to justify this course; still the opinions of others are duly respected.

We have examined the work with very great pleasure, and believe that it will prove a favorite text book in its department. It is most admirably arranged, is sufficiently comprehensive, every where bears the evidences of careful observation and mature thought, while the sound reasoning and good common sense of the author lend to it a force which is convincing. We have not space to speak in detail of any of the new views which have been presented, but assure our readers that the reasoning is sound, and the conclusions based upon adequate observation. We have no work upon the diseases of infancy and childhood which can compare with it, and we most heartily approve it as a text book for the student and as a guide to the physician.

---

Conspectus of the Medical Sciences; comprising Manuals of Anatomy, Physiology, Chemistry, Materia Medica, Practice of Medicine, Surgery and Obstetrics. By Henry Hartshorne, A. M., M. D. Philadelphia: Henry C. Lea, 1869.

This book is prepared with special view to the wants of medical students, to be used mainly while in attendance upon lectures. While we never give utterance to any very high praise of works so brief and superficial, still we must express the belief that this book will answer exactly the object of publication, and that students will find it invaluable in looking up the lectures and retaining the main facts and important points, such as all teachers insist upon students understanding most perfectly. Physicians too actively engaged in practice to consult more extensive works can also extract from it much useful knowledge.

It is illustrated in all departments with over three hundred wood cuts, and this feature adds very much to the plainness and ready teaching of the work. There is no occasion to urge such books upon the attention, the mere announcement is quite sufficient to place it in the hands of the masses of medical students who are generally quite willing to accept some ready method of obtaining a knowledge of their profession.

---

Schirrhus, or Malignant Disease of the Rectum. By Alden March, M. D.

This is a re-print from the Transactions of the New York State Medical Society for 1868, of a case reported by Prof. March of Albany, in which he removed a schirrhus mass from the rectum by operation. He made incision down upon the mass through the posterior walls of the vagina, divided the intestinal canal above and below, and removed the disease. The intestine he drew down and united it by suture, closed the incision also with sutures, introduced a metallic pile pessary into the gut and secured it with bandage. At last report of the case the patient was quite recovered, was about the house and full of hope.

Operation of Vesico-Vaginal Fistula without the aid of Assistants. By Nathan Bozeman, M. D. New York: D. Appleton & Co., 1869.

This paper was read before the New York Medical Journal Association and published in the *New York Medical Journal* for February. The author first describes and gives illustration of the manner of securing patients upon the knees and chest, so as to be able to give chloroform and not require the aid of assistants in maintaining the patient in position. The different forms of suture are compared, described and reviewed. The speculum is also illustrated and its advantages distinctly pointed out. There is considerable description of Dr. Bozeman's speculum, of his suture, of his treatment and of his results. We think him entitled to credit and great respect, and like the paper very well, it is suggestive and instructive, and all interested in the operation will be attracted by it.

---

Syphilis, and Local Contagious Disorders. By Berkly Hill, M. B., Lond., F.R.C.S. Philadelphia: Henry C. Lea, 1869.

This treatise comprises a systematic description of venereal diseases, with all the contributions which recent research in this department of medicine has brought to our knowledge. Syphilis is treated as a disease from which arises all venereal sores; the primary ulcer is not called chancre at all, but simply the primary lesion of syphilis, thus reserving the word *chancre* to designate the *local contagious ulcer* which never gives rise to constitutional disease, or shows any vitiation of the general system. Our knowledge of syphilitic disease has increased by so many new facts and the rejection of so many errors that it is now to be re-studied by all who imbibe the views entertained a few years since. "Not long ago it was impossible to describe the whole course of syphilis, for it is only recently that we have been able to assign to their true origin the numerous lesions which are now recognized as common sequences of the disease."

This work is most admirably arranged for both student and practitioner; no other work upon the subject equals it; it is more simple, more easily studied, contains what the author calls a "*summary*" added to each chapter, which embodies the whole teaching in few words. The diseases *called* venereal are separated and made distinct; the treatment is according to the best methods and sufficiently comprehensive to form a complete guide. It is certainly a book to be read by all who practice in this class of disease.

---

Some Thoughts upon Education. By John Locke. New York: J. W. Schermerhorn & Co.

This is a small volume written for everybody, and contains some very truthful and sensible remarks upon the general education of children. It should be read by all teachers and parents.

---

Report of the New York Orthopædic Dispensary.

This Dispensary is organized for the purpose of furnishing treatment to the poor with special reference to the diseases and deformities of the spine and hip-joint, and other diseases of the bones requiring mechanical treatment. The report contains statement of cases and results of treatment quite flattering to the present modes. The institution is a benevolence and will at the same time be made to advance some private interests.

Clinical Lectures upon Diseases of the Urinary Organs. By Sir Henry Thompson. Philadelphia: Henry C. Lea, 1869.

This volume, of over two hundred pages, contains twelve lectures upon the diseases of the urinary organs. The lectures are upon the following subjects: Diagnosis; Structure of the Urethra; Hypertrophy of the Prostrate and its consequences; Retention of Urine; Extravasation of Urine and its consequences and Urinary Fistulæ; Stone in the Bladder; Lithrotrity; Lithotomy; Cystitis and Prostatitis; Diseases of the Bladder; Paralysis; Stony, juvenile incontinence; Tumors; Hæmaturia and Renal Calculus.

These subjects are all considered in a familiar and attractive manner, and the lectures may be regarded as a very condensed and truthful embodiment of all that is known of practical value upon the various topics of discourse.

---

Essentials of the Principles and Practice of Medicine—A Hand-book for Students and Practitioners. By Henry Hartshorne, M. D. Second edition, revised and improved. Philadelphia: Henry C. Lea.

We have no occasion to speak of this work to any of our readers; all understand how it has received the approval of students and physicians. It is as well arranged, full, practical and useful, as any treatise can be having so wide a scope and such limited space. Its title tells just what it is, and every one may be assured that it is just what it claims to be. Students in lecture term may be forgiven for consulting it; physicians who want to learn disease with the view of controlling it, should generally select some other work, otherwise some of the "essentials" even will, after a little, be wanting.

---

On the Dynamics, Principles and Philosophy of Organic Life. How do Medicines produce their effects? By D. C. McElroy, M. D., Zanesville, Ohio.

This is the President's Valedictory Address before the Muskingum County Medical Society, Zanesville, Ohio, May 6th, 1868. It is philosophical, consistent and well considered, full of suggestion and instruction, but the scope of the argument is such that no idea can be conveyed of it without more space than we have at our command. It must be carefully read to gain any just idea of the author's meaning.

---

Fibrous Tumor removed from the Anterior Wall of the Uterus. By William H. Byford, M. D.

An extra sheet from the *Chicago Medical Examiner* contains the report of an interesting case of successful removal of fibrous tumor from the walls of the uterus. The tumor was oval in shape, weighed twenty ounces, was five inches and a half long, four inches and three-quarters broad and four and a quarter thick. "An incision was first made in the most dependent part of the tumor, in the anterior lip of the uterus, which extended transversely from one side of the pelvis to the other, and must have been over three inches long;" another was extended up the posterior of the tumor over three inches. The tumor thus reached was enucleated, and extracted from its bed. The patient recovered without unfavorable symptoms.

---

Report on Insanity. By Charles A. Lee, Peekskill, N. Y. (Extracted from the Transactions of the American Medical Association.)

Prof. Lee speaks of the number of insane, the causes of insanity, and then gives brief history of the Insane Hospitals of the United States. The report embodies a great amount of statistical matter, and also instructive suggestions as to the best methods of caring for the insane. Prof. Lee has studied this subject from all points of observation, both in this and foreign countries, and no one is better prepared to present the important subjects connected with it, than is the author of this report.

---

Catalogue of Surgical Instruments manufactured by W. F. Ford, (late Wade & Ford) 85 Fulton street, New York, Instrument Maker to the N. Y. City, Bellevue and N. Y. S. Woman's Hospitals.

This catalogue contains detail description of contents of various cases and sets, and the price of all instruments used by surgeons, physicians and specialists. Those proposing to purchase can obtain by application this descriptive catalogue, and from it order goods almost as well as if present to make personal selections. Microscopes, Ophthalmoscopes, Laryngoscopes, and indeed *all* instruments in use by the profession are for sale by Mr. Ford, and will be found described in the catalogue with the price appended.

